# Progress in Laryngology

**Published:** 1895-10-26

**Authors:** 


### PROGRESS IN LARYNGOLOGY.

(*Continued from page* 50.)

**Laryngeal Lesions of Typhoid.**—Dr. Lucatello[18] has made experimental researches which show that these are attributable exclusively to the specific microbe of that disease. Like other pyogenic organisms they are capable of setting up abscess formation as a result of secondary infection of the parts.

**Pneumothorax Following the Passage of a Plumstone into the Larynx.**—A case is described by Dr. Carslaw.[19] Tracheotomy was performed as a precautionary measure two days after the stone had been swallowed, but not till a month later did it make its appearance, when it was coughed up *per vias naturales*. The patient recovered with slight collapse of the left chest wall. The cause of the pneumothorax was not apparent.

**Vocal Breakdown on the part of Board School Teachers.**—At a recent meeting of the British Laryngological Association,[20] this subject was discussed on the occasion of a succession of cases of myopathic aphonia that had been exhibited. The arytenoideus in one case, and the internal thyro-arytenoid in another (shown by Dr. Dundas Grant), were the muscles specially involved. It was suggested that the society should memorialise the authorities on this subject, Board School teachers being in the meantime instructed as to the proper methods of training and producing the voice when coming up for advice.

**Chronic Laryngitis.**—Dr. Krause[21] recommends scarification of the congested mucous membrane in certain cases. For an excellent *résumé* of the whole treatment of this intractable malady the reader is referred to an excellent clinical lecture by Dr. Greville Macdonald.[22]

**Sodium Benzoate** is given by Liegos[23] in acute laryngitis and pharyngitis. The dose for a child is 75 grains per day, that for an adult from 150 to 225.

**Structure of so-called Fibroma of the Vocal Cord.**—Chiari, in an elaborate paper read before the Rome Congress, holds the opinion that the true fibroma is probably rare, most of the growths thus designated being, in fact, circumscribed hypertrophies of all the superficial layers of the vocal cord, *i.e.*, a polypus in Eppinger's sense, and not true fibromata. He considers that their origin in chronic inflammatory thickening of the cords has been proved both by serial section cutting of the cord and growth together, and by clinical observation.

[18] Amer. Jour. of Med. Sci., Jan., 1895. [19] Glasg. Med. Journ., April 18, 1895. [20] Journ. of Lar., Rhin., and Otol., March, 1895. [21] Berlin Klinik Wochen Schr., No. 16, 1894. [22] Internat. Clinics, iii., Vol. IV. [23] Deut. Med. Wochen, 1895.

